# Adverse Events Following Immunization Associated with the First and Second Doses of the ChAdOx1 nCoV-19 Vaccine among Healthcare Workers in Korea

**DOI:** 10.3390/vaccines9101096

**Published:** 2021-09-28

**Authors:** Minji Jeon, Jehun Kim, Chi Eun Oh, Jin-Young Lee

**Affiliations:** 1 Division of Infectious Diseases, Department of Internal Medicine, Kosin University Gospel Hospital, Kosin University College of Medicine, Busan 49267, Korea; thehatter57@gmail.com; 2 Division of Pulmonary and Critical Care Medicine, Department of Internal Medicine, Kosin University Gospel Hospital, Kosin University College of Medicine, Busan 49267, Korea; libertier@gmail.com; 3 Department of Pediatrics, Kosin University Gospel Hospital, Kosin University College of Medicine, Busan 49267, Korea; chieunoh@kosin.ac.kr

**Keywords:** COVID-19, vaccination, ChAdOx1 nCoV-19 vaccine, adverse events following immunization

## Abstract

As worldwide large-scale inoculation of novel vaccines is on the way, the importance of real-world data on safety cannot be overemphasized. We aimed to investigate the adverse events following immunization (AEFIs) associated with the ChAdOx1 nCoV-19 vaccine among healthcare workers (HCWs). We investigated the systemic and local adverse events reported within seven days following the first and second doses of vaccination, using the mobile vaccine adverse events reporting system (MVAERS) developed by our hospital. The response rates were 71.8% (994/1384) and 52.9% (727/1375) after the first and second doses, respectively. The most commonly reported AEFIs were tenderness and pain at the injection site and fatigue after the first and second doses. In comparison to the first dose, the incidence and severity of AEFIs were lower following the second dose. Since the Korean government does not recommend the ChAdOx1 nCoV-19 vaccination for those under 30 years of age, with greater risk than benefit, we additionally compared the AEFIs of age groups under and above 30 years of age. The overall incidence of AEFIs was similar in both the under and over 30 age groups. In conclusion, AEFIs associated with the ChAdOx1 nCoV-19 vaccine were found to be tolerable, and AEFIs associated with the second dose were less common and severe compared to the first dose. Further safety surveillance studies on COVID-19 vaccines are required to validate our findings.

## 1. Introduction

Globally, the coronavirus disease 2019 (COVID-19) has been reported have a morbidity of more than 170 million patients and a mortality of more than 3 million as of June 2021 [1]. Antiviral drugs (e.g., remdesivir) and immune modulators (e.g., tocilizumab and baricitinib) are being tried as therapeutic agents; however, the most important way to overcome the current pandemic is large-scale vaccination worldwide. Vaccination policies differ from country to country, but in some countries, vaccination is still not available or is preferentially administered.

In the Republic of Korea, COVID-19 vaccination has been initiated in February 2021, with a total of 15,279,057 people having received either one or more vaccine doses as of 26 June 2021 [2]. Notably, frontline healthcare workers (HCWs) were given vaccination priority, with the allocation of BNT162b2 mRNA vaccines (Pfizer/BioNTech) or the ChAdOx1 nCoV-19 vaccines (AstraZeneca). Although randomized controlled trials have demonstrated the efficacy and safety of the ChAdOx1 nCoV-19 vaccine [3,4], several cases of severe adverse events associated with this vaccine have also been reported [5,6]. Poor dissemination of medical information and concerns regarding the safety and efficacy of rapidly developed novel vaccines are the factors that lead to vaccine hesitancy [7]. Moreover, since many people are easily exposed to disinformation about vaccination and treatment of COVID-19 when using the internet or social media, evidence-based accurate information should be shared seamlessly worldwide.

To consistently achieve large-scale inoculation and provide precise information on novel vaccines against COVID-19, research on the incidence and severity of adverse events in real-world settings is needed. Therefore, we aimed to evaluate the incidence and severity of adverse events following immunization (AEFIs) associated with the ChAdOx1 nCoV-19 vaccine and to investigate the differences in AEFIs between the first and second vaccine doses among HCWs at a single university hospital. Specifically, we used the medical records of AEFIs among HCWs, which were voluntarily reported in the mobile vaccine adverse events reporting system (MVAERS) developed by our hospital.

## 2. Materials and Methods

### 2.1. Study Design and Population

This was a retrospective, single-center cohort study conducted at a teaching hospital in the Republic of Korea, and the study subjects were HCWs who had received at least one dose of the ChAdOx1 nCoV-19 vaccine. Since the Korean government recommended the ChAdOx1 nCoV-19 vaccine for those under 65 years of age initially, only HCWs under the age of 65 years were included in this study. Subsequently, following the reports of serious adverse events associated with the ChAdOx1 nCoV-19 vaccine [5,6,8], the Korean government has also banned its vaccination for those under 30 years of age; however, it was possible to voluntarily proceed with the second dose for HCWs under the age of 30, if they had already received the first dose and did not have any serious adverse events at that time [9]. For those who consented to undergo vaccination with the second dose, the vaccine was administered at an interval of 11 weeks. Among those who received the vaccine, HCWs who did not report to the MVAERS or had retired from our hospital before the end of the monitoring period after the second dose were excluded.

### 2.2. Adverse Events Reporting System

We developed a mobile web page, named “MVAERS,” to systematically capture spontaneous reporting of AEFIs for their monitoring and management, which was used to collect the data that were analyzed in this study. In brief, all vaccinated HCWs received webpage hyperlinks by a short message system twice a day for seven days from the day of vaccination, in which AEFIs were voluntarily reported using a questionnaire inquiring about the 12 adverse events of local and systemic AEFIs. Solicited local AEFIs included tenderness, pain at rest, redness, and swelling at the injection site, whereas solicited systemic AEFIs included fatigue, headache, malaise, arthralgia, chills, fever, nausea or vomiting, and diarrhea. Additionally, the severity of the local and systemic AEFIs was graded on a scale of 1 to 4 based on the guidelines of the Korean Ministry of Food and Drug Safety and the U.S. Food and Drug Administration (Supplementary Table S1) [10].

### 2.3. Statistical Analysis

Categorical variables were compared using Pearson’s χ^2^ test, continuous variables were compared using the Student’s t-test or the Mann-Whitney test, and the severity of AEFIs between the first and second doses were compared using the Wilcoxon signed-rank test. Data of the AEFIs after the first dose from our previous study were used in the current study [11]. Statistical significance was set at *p* < 0.05, and statistical analyses were performed using the SPSS software (version 25.0; IBM Corp., Armonk, NY, USA).

## 3. Results

A total of 1954 HCWs were employed at the end of monitoring after second dose administration. Among them, a total of 1384 HCWs were administered the first vaccine dose, wherein 994 of them (71.8%) were reported in the MVAERS. The mean age of the respondents was 35.7 years (range: 19–63 years), and 76.7% were female. Meanwhile, a total of 1375 HCWs received the second vaccine dose, wherein the data of 727 of them (52.9%) were reported in the MVAERS. The mean age of the respondents for this group was 36.7 years (range: 20–63 years), and 76.9% were female. When the confidence level was 95%, the sample distribution had a 2.2% and 2.8% margin of error after the first and second dose vaccination, respectively.

The incidence and severity of AEFIs associated with the first and second doses of the ChAdOx1 nCoV-19 vaccine are shown in Table 1. After first dose administration, 975 (98.1%) reported more than one AEFI, and the incidence of local AEFIs was similar to that of systemic AEFIs (95.4% vs. 95.1%, respectively). The most commonly reported systemic AEFIs were fatigue (923, 92.9%) and malaise (833, 83.8%), whereas the most commonly reported local AEFIs were resting pain (871, 87.6%) and tenderness (608, 61.2%) at the injection site. On the other hand, after second dose administration, 661 (90.9%) reported more than one AEFI, and the incidence of local AEFIs was higher than that of systemic AEFIs (84.2% vs. 75.5%, respectively). The most commonly reported AEFIs for this group were tenderness at the injection site (602, 82.8%), fatigue (505, 69.5%), and resting pain at the injection site (481, 66.2%).

**Figure 1:**
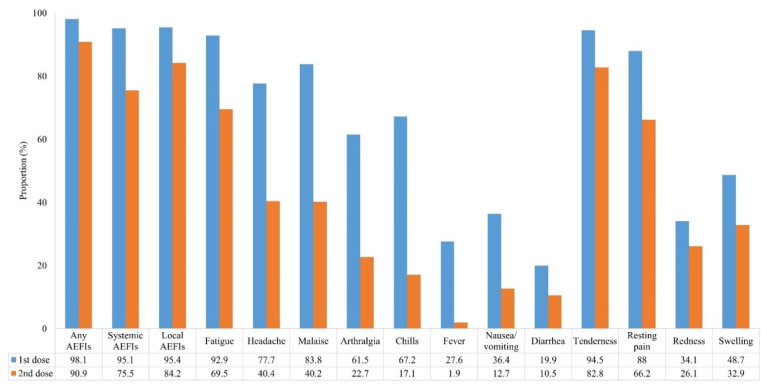
Incidence of adverse events following the administration of the first and second ChAdOx1 nCoV-19 vaccine doses. The numbers are presented as rate (%). (*p* < 0.001 for all variables).

**Figure 2:**
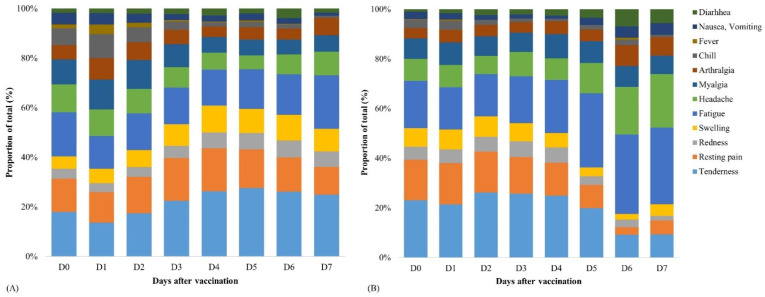
Distribution of the types of adverse events after administering the first dose (**A**) and second dose (**B**) of the ChAdOx1 nCoV-19 vaccine.

The incidence of AEFIs after the administration of the first and second doses of ChAdOx1 nCoV-19 vaccine is shown in Figure 1. As compared to the first dose, the incidence of any AEFIs was lower after the second dose (98.1% vs. 90.9%, *p* < 0.001). In particular, systemic AEFIs were significantly fewer after the second dose than after the first dose. The distribution of the types of AEFIs after administration of the first and second ChAdOx1 nCoV-19 vaccine doses is illustrated in Figure 2. It was found that local AEFIs accounted for approximately 40% of the reported AEFIs within seven days after the first dose administration. In contrast, as time passed after the second dose administration, the proportion of local AEFIs decreased, while the proportion of fatigue and headache increased.

**Figure 3:**
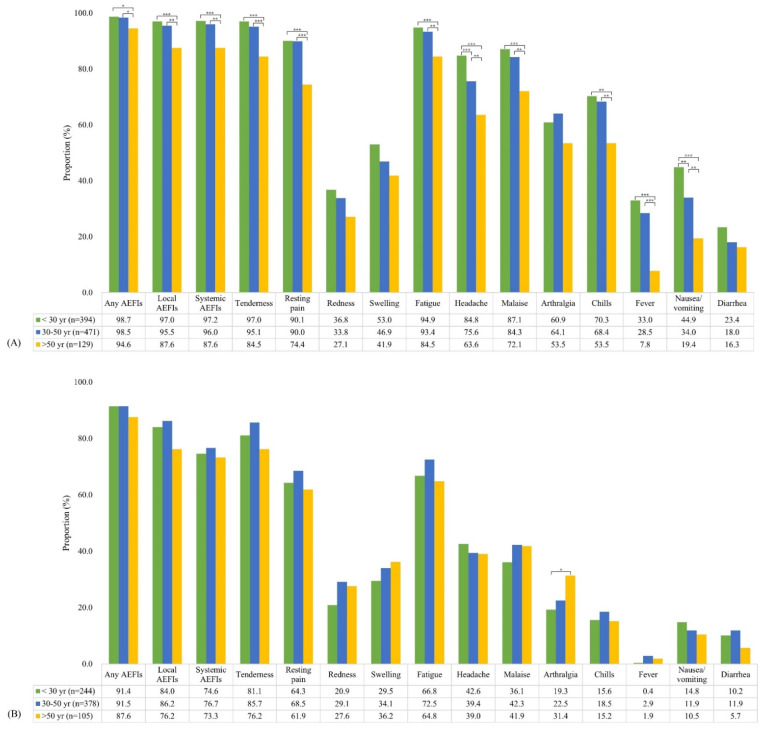
Incidence of adverse events after administering the first dose (**A**) and second dose (**B**) of the ChAdOx1 nCoV-19 vaccine stratified by age. (* *p* < 0.05, ** *p* < 0.01, *** *p* < 0.001).

The incidence of AEFIs by age group is summarized in Table 2 and Figure 3. After first dose administration, AEFIs showed a significant decreasing trend with age, with the exception of redness at the injection site and arthralgia. Moreover, all AEFIs were reported to be more severe in younger HCWs than in older HCWs (Supplementary Table S2). However, after second dose administration, there were no significant differences in the incidence of AEFIs according to age groups, with the exception of arthralgia (*p* for trend = 0.020).

Additionally, we analyzed the paired data of the 652 HCWs who reported AEFIs after the first and second doses (652/1375, 47.4%), showing that the incidence and severity of all AEFIs after the second dose were significantly lower than those after the first dose (Table 3). Similar to the overall cohort, there was also no correlation in the incidence of AEFIs according to age group, with the exception of arthralgia (data not shown).

In accordance with the government’s policy not to recommend ChAdOx1 nCoV-19 vaccination for those under 30 years of age, who were evaluated as having a greater risk than benefit, only HCWs under 30 years of age who did not have severe AEFIs after the first dose voluntarily received a second dose. In our hospital, 480 HCWs under 30 years of age were administered the first dose, while only 459 HCWs received the second dose. Among them, 217 HCWs reported at least one AEFI after receiving both the first and second doses (response rate: 217/459, 47.3%), with no serious events requiring hospitalization observed among HCWs under 30 years of age. In paired data analysis from the 217 HCWs under 30 years of age, the incidence and severity of AEFIs after the second dose were found to be significantly lower than those after the first dose (Table 4, *p* < 0.001 for all).

On comparison between the two age groups, both the incidence of overall AEFIs after the first and second doses was similar between those under 30 years of age and those over 30 years of age (Supplementary Table S3). However, in terms of individual categories, local AEFIs, including tenderness and swelling at the injection site, and systemic AEFIs, including headache, malaise, fever, nausea or vomiting, and diarrhea, were more frequent in HCWs under 30 years of age after first dose administration. Meanwhile, after second dose administration, only redness at the injection site was more frequently reported in HCWs over 30 years of age than in those under 30 years of age (28.8% vs. 20.9%, *p* = 0.028).

We summarized the information and chief complaints of HCWs who visited the emergency room (ER) or outpatient clinic at the Infectious Diseases Department (ID) in Supplementary Table S4. After first dose administration, 13 HCWs visited the ER or outpatient clinic at the ID, presenting with chief complaints of urticaria, pain at the injection site, fever, chills, malaise, headache, dizziness, diarrhea, tingling sensation, and weakness of the lower extremities. One HCW, in particular, was immediately referred to the ER due to dyspnea, nausea, and hypotension within 10 min after vaccination, but the HCW rapidly recovered and was discharged after supportive treatment. On the other hand, after second dose administration, 5 HCWs visited the outpatient clinic at the ID, presenting with chief complaints of arthralgia and a tingling sensation in both legs, throat pain, and fatigue, all of which improved spontaneously within a few days. In particular, only one HCW, who did not report any AEFIs to MVAERS, complained of multiple bruises in both arms, and visited the outpatient clinic seven days after receiving the second dose. On initial consultation, the HCW refused any further evaluation despite being warned. Five days later, he revisited the outpatient clinic and underwent laboratory tests, showing a platelet count of 149 × 10^3^/µL and the d-dimer level of 0.26 µg/mL. He then refused brain imaging, since his symptoms improved spontaneously without any intervention, and is currently under close follow-up.

## 4. Discussion

In this study, we found that at least one AEFI was reported in more than 90% of HCWs who received the ChAdOx1 nCoV-19 vaccine. As compared to the first dose administration, the incidence and severity of AEFIs after the second dose were reported to be lower. Moreover, after first dose vaccination, the incidence of most AEFIs showed a decreasing trend with age; however, there was no significant trend between incidence and age following second dose vaccination. Furthermore, no serious AEFIs requiring hospitalization or death were reported during the monitoring period after the first and second dose vaccinations.

With the implementation of large-scale vaccinations worldwide, AEFIs associated with the ChAdOx1 nCoV-19 vaccine have been reported in several countries, with varying incidences between recent studies [12,13,14,15,16]. However, similar to our findings, fatigue, malaise, tenderness, and pain at the injection site were the commonly reported AEFIs after the first dose of vaccination in several studies [12,13,14,15,16]. As compared to previous studies of AEFIs associated with the first dose of the ChAdOx1 nCoV-19 vaccine in Korea [12,16], the incidence of local and systemic AEFIs after the first vaccination was higher in our study. Particularly, a study by Kim et al., which performed a multicenter survey in referral teaching hospitals, reported that injection site tenderness (69.6%) and malaise (74.6%) were the most commonly reported AEFIs [12]. Similarly, in another study by Kim et al. performed in a tertiary hospital, they reported that injection site pain (77.8%) and malaise (60.5%) were the most commonly reported AEFIs [16]. Since the demographics of the study population were similar, this may have been influenced by the subjectivity of self-reported symptoms caused by the nature of the survey.

We reported that the incidence and severity of AEFIs after the second dose were lower than those after the first dose, which were consistent with the results of the ChAdOx1 nCoV-19 vaccine clinical trials [4,17], reporting that the reactogenicity profile after the second dose appeared to be less severe. As of 8 August 2021, a total of 11,563,991 ChAdOx1 nCoV-19 vaccine doses were inoculated in Korea, and the total number of reported AEFIs was 78,058. Among them, the incidence of AEFIs after the second dose was reported to be lower than that after the first dose (0.72% vs. 0.22%, respectively) [18]. These findings may lead to improved vaccination rates for those who are reluctant to receive the second dose due to experiencing adverse events after the first ChAdOx1 nCoV-19 vaccine dose. In our study, unlike the first dose vaccination, there was no significant trend between incidence and age following the second dose vaccination. Previous studies on AEFIs showed that the incidence of AEFIs tended to decrease with increasing age after first dose administration [12,13,16]. However, since these studies do not deal with AEFIs after the second dose, additional research is needed on the correlation between the AEFIs after the second dose and age in real-world settings.

As of 11 April 2021, the Korean government has not allowed the administration of the ChAdOx1 nCoV-19 vaccine for those under 30 years of age who were evaluated as having a greater risk than benefit [9]. Therefore, we additionally analyzed the differences in the incidence and severity of AEFIs between HCWs under 30 years of age and those over 30 years of age. On comparison, we found that the incidence of overall AEFIs was similar between the two groups after the first and second doses. However, tenderness and swelling at the injection site, headache, malaise, fever, nausea or vomiting, and diarrhea were frequently reported to occur after the first dose in the under 30 years of age group. Meanwhile, after second dose administration, only redness at the injection site was more frequently reported in the over 30 years age group. These findings support the Korean vaccination policy for the allowance of select individuals under the age of 30 who have already received the first ChAdOx1 nCoV-19 vaccine dose to receive the second dose.

In our study, there were no reports of serious adverse events, except for one case of thrombocytopenia, which spontaneously recovered within a few days. By 8 August 2021, 11.56 million doses of the ChAdOx1 nCoV-19 vaccine were administered in Korea, and 78,058 adverse events were reported. The incidence of severe adverse events was 0.03% (3109/11,563,991): encephalopathy, 223 (19.3 per million); Guillain Barre Syndrome, 104 (9.0 per million); thrombocytopenic purpura, 787 (68.1 per million); and anaphylaxis, 78 (6.7 per million) [18]. In particular, only two cases of thrombosis with thrombocytopenia syndrome (TTS) were reported (0.2 per million) [18]. In particular, only two cases of thrombosis with thrombocytopenia syndrome (TTS) were reported (0.2 per million). As a result, to reflect the risk of this fatal adverse event, the ChAdOx1 nCoV-19 vaccination policy was revised to be recommended for those aged 50 and over as of July 2021 [19]. On the other hand, 803 cases of anaphylaxis and 412 cases of TTS were reported in the United Kingdom (administration: 38.5 million doses as at 4 August 2021) and 55 cases of anaphylaxis and 157 cases of TTS were reported in the Germany (administration: 11.5 million doses until 30 June 2021) [20,21]. This is significantly higher than the results of an interim analysis of four clinical trials on the ChAdOx1 nCoV-19 vaccine, which reported that the incidence of thromboembolic events, including coronary artery occlusion, ischemic stroke, pulmonary embolism, and thrombosis, was less than 0.1% [17]. The difference in the incidence of severe adverse events across countries may be attributed to differences in the total number of vaccinations or racial differences. In terms of vaccine hesitancy, medical education or contents of mass media that reinforce confidence in the safety of novel vaccines may have led to a shift toward vaccine acceptance [22,23]. Therefore, we considered our findings to be quite important, because they support the fact that the incidence of severe adverse events is not very high. Our findings will contribute to improving vaccination rates globally.

Notably, Cheng et al.’s meta-analysis reported that all of the COVID-19 vaccines that have published their phase III clinical trials data did not increase the risk of serious adverse events [24]. Despite this, a number of important adverse events reported in real-world settings require careful evaluation and monitoring. Although several previous studies on adverse events associated with the ChAdOx1 nCoV-19 vaccine have been published, these studies included populations only after the first dose or a lower proportion of second dose recipients. In contrast, to date and to the best of our knowledge, this is the first study to compare the incidence and severity of AEFIs after the first and second ChAdOx1 nCoV-19 vaccine doses using paired data analysis. Thus, we believe that our study results provided more information on the adverse events associated with the ChAdOx1 nCoV-19 vaccine, especially after second dose administration.

Despite these findings, our study has several limitations. First, the AEFIs were not objectively reported due to the design of the voluntary reporting system. Additionally, AEFIs that were not included in the questionnaire were not routinely monitored. Second, the results might not be generalizable since this study was conducted at a single center, comprising subjects younger than 65 years of age. And the sample size was relatively small, and there were unresponded data that could cause bias. Large-scale, long-term safety monitoring research is required in the future. Third, the response rate after the second dose vaccination was lower than that after the first dose vaccination, thus the possibility of underestimation cannot be excluded. Fourth, we cannot rule out the possibility of unobserved bias caused by the occupational status of the participants. Medical professionals might do a better job of self-reporting on their symptoms; however, the reported AEFIs could be underestimated or overestimated. Finally, further studies are required to compare the AEFIs of other novel vaccines. During the study period, the vaccine that was first supplied to healthcare workers in our hospital was limited to the ChAdOx1 nCoV-19 vaccine. Reviewing other vaccines can provide reasonable safety results.

In conclusion, fatigue, pain, and tenderness at the injection site were frequently reported after ChAdOx1 nCoV-19 vaccination. On comparison, the incidence and severity of AEFIs after the second dose were lower than those after the first dose. Moreover, following second dose administration, there was no difference in the incidence of AEFIs according to age, unlike after the first dose. The overall incidence of AEFIs in patients under 30 years of age, who were evaluated as having a greater risk than benefit for vaccination, was similar to the incidence for those over 30 years of age. Given the limitations of this study, further safety surveillance studies of COVID-19 vaccines are required. Safe vaccination can be promoted through active monitoring of adverse events through various methods such as MVAERS and further investigation of safety.

## Figures and Tables

**Table 1:** Incidence and severity of adverse events following the first and second doses of the ChAdOx1n CoV-19 vaccine.

Adverse Events	1st Dose (*n* = 994)						2nd Dose (*n* = 727)						*p* Value
	Any	None	G1	G2	G3	G4 ^a^	Any	None	G1	G2	G3	G4 ^a^	
Age, mean ± SD	35.66 ± 10.46 (range, 19–63)						36.75 ± 10.45 (range, 20–63)						0.034
Female sex	762 (76.7)						559 (76.9%)						0.911
Any AEFIs	975 (98.1)	19 (1.9)	108 (10.9)	422 (42.5)	428 (43.1)	17 (1.7)	661 (90.9)	66 (9.1)	221 (30.4)	303 (41.7)	134 (18.4)	3 (0.4)	<0.001
Local AEFIs	948 (95.4)	46 (4.6)	189 (19.0)	547 (55.0)	207 (20.8)	5 0.5)	612 (84.2)	115 (15.8)	242 (33.3)	252 (34.7)	115 (15.8)	3 (0.4)	<0.001
Tenderness	939 (94.5)	55 (5.5)	208 (20.9)	400 (40.2)	331 (33.3)		602 (82.8)	125 (17.2)	240 (33.0)	250 (34.4)	112 (15.4)		<0.001
Resting pain	875 (88.0)	119 (12.0)	543 (54.6)	328 (33.0)	4 (0.4)		481 (66.2)	246 (33.8)	413 (56.8)	68 (9.4)	0 (0.0)		<0.001
Redness	339 (34.1)	655 (65.9)	251 (25.3)	49 (4.9)	31 (3.1)	8 (0.8)	190 (26.1)	537 (73.9)	172 (23.7)	12 (1.7)	5 (0.7)	1 (0.1)	<0.001
Swelling	484 (48.7)	510 (51.3)	326 (32.8)	108 (10.9)	40 (4.0)	10 (1.0)	239 (32.9)	488 (67.1)	186 (25.6)	42 (5.8)	8 (1.1)	3 (0.4)	<0.001
Systemic AEFIs	945 (95.1)	49 (4.9)	191 (19.2)	405 (40.7)	337 (33.9)	12 (1.2)	549 (75.5)	178 (24.5)	294 (40.4)	223 (30.7)	32 (4.4)	0 (0.0)	<0.001
Fatigue	923 (92.9)	71 (7.1)	276 (27.8)	454 (45.7)	193 (19.4)		505 (69.5)	222 (30.5)	310 (42.6)	165 (22.7)	30 (4.1)		<0.001
Headache	772 (77.7)	222 (22.3)	262 (26.4)	502 (50.5)	8 (0.8)		294 (40.4)	433 (59.6)	175 (24.1)	118 (16.2)	1 (0.1)		<0.001
Malaise	833 (83.8)	161 (16.2)	236 (23.7)	580 (58.4)	17 (1.7)		292 (40.2)	435 (59.8)	196 (27.0)	94 (12.9)	2 (0.3)		<0.001
Arthralgia	611 (61.5)	383 (38.5)	227 (22.8)	377 (37.9)	7 (0.7)		165 (22.7)	562 (77.3)	114 (15.7)	51 (7.0)	0 (0.0)		<0.001
Chills	668 (67.2)	326 (32.8)	232 (23.3)	421 (42.4)	15 (1.5)		124 (17.1)	603 (82.9)	86 (11.8)	35 (4.8)	3(0.4)		<0.001
Fever	274 27.6)	720 (72.4)	181 (18.2)	74 (7.4)	17 (1.7)	2 (0.2)	14 (1.9)	713 (98.1)	11 (1.5)	2 (0.3)	1 (0.1)	0 (0.0)	<0.001
Nausea/ vomiting	362 (36.4)	632 (63.6)	288 (29.0)	67 (6.7)	7 (0.7)		92 (12.7)	635 (87.3)	84 (11.6)	6 (0.8)	2 (0.3)		<0.001
Diarrhea	198 19.9)	796 (80.1)	119 (12.0)	66 (6.6)	10 (1.0)	3 (0.3)	76 (10.5)	651 (89.5)	43 (5.9)	29 (4.0)	4 (0.6)	0 (0.0)	<0.001

Data are shown as number (%). AEFI = adverse event following immunization; G = Grade. ^a^ Fever, diarrhea, redness, and swelling at the injection site were included in Grade 4 severity.

**Table 2:** Incidence of adverse events following the first and second ChAdOx1 nCoV-19 vaccine doses by age group.

	1st Dose				2nd Dose			
Adverse Events	<30 Years (*n* = 394)	30–50 Years (*n* = 471)	>50 Years (*n* = 129)	*p* Value for Trend	<30 Years (*n* = 244)	30–50 Years (*n* = 378)	>50 Years (*n* = 105)	*p* Value for Trend
Any AEFIs	389 (98.7)	464 (98.5)	122 (94.6)	0.017	223 (91.4)	346 (91.5)	92 (87.6)	0.384
Local AEFIs	382 (97.0)	450 (95.5)	113 (87.6)	<0.001	205 (84.0)	326 (86.2)	81 (77.1)	0.321
Tenderness	382 (97.0)	448 (95.1)	109 (84.5)	<0.001	198 (81.1)	324 (85.7)	80 (76.2)	0.712
Resting pain	355 (90.1)	424 (90.0)	96 (74.4)	<0.001	157 (64.3)	259 (68.5)	65 (61.9)	>0.999
Redness	145 (36.8)	159 (33.8)	35 (27.1)	0.053	51 (20.9)	110 (29.1)	29 (27.6)	0.076
Swelling	209 (53.0)	221 (46.9)	54 (41.9)	0.016	72 (29.5)	129 (34.1)	38 (36.2)	0.173
Systemic AEFIs	383 (97.2)	452 (96.0)	113 (87.6)	<0.001	182 (74.6)	290 (76.7)	77 (73.3)	>0.999
Fatigue	374 (94.9)	440 (93.4)	109(84.5)	0.001	163 (66.8)	274 (72.5)	68 (64.8)	0.856
Headache	334 (84.8)	356 (75.6)	82 (63.6)	<0.001	104 (42.6)	149 (39.4)	41 (39.0)	0.461
Malaise	343 (87.1)	397 (84.3)	93 (72.1)	<0.001	88 (36.1)	160 (42.3)	44 (41.9)	0.192
Arthralgia	240 (60.9)	302 (64.1)	69 (53.5)	0.440	47 (19.3)	85 (22.5)	33 (31.4)	0.020
Chills	277 (70.3)	322 (68.4)	69 (53.5)	0.003	38 (15.6)	70 (18.5)	16 (15.2)	0.825
Fever	130 (33.0)	134 (28.5)	10 (7.8)	<0.001	1 (0.4)	11 (2.9)	2 (1.9)	0.158
Nausea/vomiting	177 (44.9)	160 (34.0)	25 (19.4)	<0.001	36 (14.8)	45 (11.9)	11 (10.5)	0.241
Diarrhea	92 (23.4)	85 (18.0)	21 (16.3)	0.034	25 (10.2)	45 (11.9)	6 (5.7)	0.467

Values are presented as number (%). AEFI = adverse event following immunization.

**Table 3:** Incidence and severity of the adverse events following the first and second ChAdOx1 nCoV-19 vaccine doses in paired data.

Adverse Events	1st Dose (*n* = 652)						2nd Dose (*n* = 652)						*p* Value ^b^
	Any	None	G1	G2	G3	G4 ^a^	Any	None	G1	G2	G3	G4 ^a^	
Age, mean ± SD	36.75 ± 10.41 (range, 20–63)												
Female sex	513 (78.7)												
Any AEFIs	642 (98.5)	10 (1.5)	82 (12.6)	256 (39.3)	293 (44.9)	11 (1.7)	598 (91.7)	54 (8.3)	194 (29.8)	275 (42.2)	127 (19.5)	2 (0.3)	<0.001
Local AEFIs	626 (96.0)	26 (4.0)	132 (20.2)	252 (38.7)	234 (35.9)	8 (1.2)	557 (85.4)	95 (14.6)	213 (32.7)	232 (35.6)	110 (16.9)	2 (0.3)	<0.001
Tenderness	622 (95.4)	30 (4.6)	144 (22.1)	251 (38.5)	227 (34.8)		548 (84.0)	104 (16.0)	212 (32.5)	229 (35.1)	107 (16.4)		<0.001
Resting pain	583 (89.4)	69 (10.6)	355 (54.4)	226 (34.7)	2 (0.3)		445 (68.3)	207 (31.7)	380 (58.3)	65 (10.0)	0 (0.0)		<0.001
Redness	222 (34.0)	430 (66.0)	159 (24.4)	35 (5.4)	23 (3.5)	5 (0.8)	177 (27.1)	475 (72.9)	161 (24.7)	12 (1.8)	4 (0.6)	0 (0.0)	<0.001
Swelling	327 (50.2)	325 (49.8)	216 (33.1)	73 (11.2)	31 (4.8)	7 (1.1)	223 (34.2)	429 (65.8)	173 (26.5)	42 (6.4)	6 (0.9)	2 (0.3)	<0.001
Systemic AEFIs	621 (95.2)	31 (4.8)	130 (19.9)	354 (54.3)	134 (20.6)	3 (0.5)	497 (76.2)	155 (23.8)	265 (40.6)	202 (31.0)	30 (4.6)	0 (0.0)	<0.001
Fatigue	604 (92.6)	48 (7.4)	178 (27.3)	301 (46.2)	125 (19.2)		458 (70.2)	194 (29.8)	282 (43.3)	148 (22.7)	28 (4.3)		<0.001
Headache	504 (77.3)	148 (22.7)	175 (26.8)	323 (49.5)	6 (0.9)		271 (41.6)	381 (58.4)	161 (24.7)	109 (16.7)	1 (0.2)		<0.001
Malaise	542 (83.1)	110 (16.9)	156 (23.9)	373 (57.2)	13 (2.0)		262 (40.2)	390 (59.8)	177 (27.1)	83 (12.7)	2 (0.3)		<0.001
Arthralgia	395 (60.6)	257 (39.4)	151 (23.2)	240 (36.8)	4 (0.6)		150 (23.0)	502 (77.0)	103 (15.8)	47 (7.2)	0 (0.0)		<0.001
Chills	418 (64.1)	243 (35.9)	138 (21.2)	271 (41.6)	9 (1.4)		112 (17.2)	540 (82.8)	75 (11.5)	34 (5.2)	3 (0.5)		<0.001
Fever	171 (26.2)	481 (73.8)	116 (17.8)	41 (6.3)	12 (1.8)	2 (0.3)	12 (1.8)	640 (98.2)	10 (1.5)	1 (0.2)	1 (0.2)	0 (0.0)	<0.001
Nausea/vomiting	226 (34.7)	426 (65.3)	185 (28.4)	36 (5.5)	5 (0.8)		86 (13.2)	566 (86.8)	79 (12.1)	5 (0.8)	2 (0.3)		<0.001
Diarrhea	127 (19.5)	525 (80.5)	82 (12.6)	39 (6.0)	5 (0.8)	1 (0.2)	70 (10.7)	582 (89.3)	41 (6.3)	27 (4.1)	2 (0.3)	0 (0.0)	<0.001

Data are presented as number (%). AEFI = adverse event following immunization; G = Grade. ^a^ Fever, diarrhea, redness, and swelling at the injection site were included in Grade 4 severity. ^b^ *p* value analyzed by Wilcoxon signed rank test.

**Table 4:** Incidence and peak grade of adverse events following the first and the second ChAdOx1 nCoV-19 vaccine doses of healthcare workers under 30 years of age in paired data.

Adverse Events	1st Dose (*n* = 217)		2nd Dose (*n* = 217)		*p* Value ^a^
Age, Mean ± SD	26.1 ± 2.1 (Range, 20–29)				
Female sex	192 (88.5)				
	Incidence	Peak grade	Incidence	Peak grade	
Any AEFIs	215 (99.1)	3.5 ± 0.7	202 (93.1)	2.8 ± 0.8	<0.001
Local AEFIs	212 (97.7)	3.3 ± 0.8	185 (85.3)	2.5 ± 0.9	<0.001
Tenderness	212 (97.7)	3.2 ± 0.8	179 (82.5)	2.5 ± 0.9	<0.001
Resting pain	196 (90.3)	2.3 ± 0.6	143 (65.9)	1.7 ± 0.6	<0.001
Redness	80 (36.9)	1.5 ± 0.9	48 (22.1)	1.2 ± 0.5	<0.001
Swelling	117 (53.9)	1.8 ± 1.0	69 (31.8)	1.4 ± 0.7	<0.001
Systemic AEFIs	212 (97.7)	3.1 ± 0.7	165 (76.0)	2.2 ± 0.8	<0.001
Fatigue	206 (94.9)	2.9 ± 0.8	148 (68.2)	2.0 ± 0.8	<0.001
Headache	186 (85.7)	2.5 ± 0.7	94 (43.3)	1.6 ± 0.8	<0.001
Malaise	190 (87.6)	2.5 ± 0.7	80 (36.9)	1.5 ± 0.7	<0.001
Arthralgia	126 (58.1)	2.0 ± 0.9	42 (19.4)	1.3 ± 0.6	<0.001
Chills	146 (67.3)	2.2 ± 0.9	34 (15.7)	1.2 ± 0.6	<0.001
Fever	73 (33.6)	1.5 ± 0.7	1 (0.5)	1.0 ± 0.1	<0.001
Nausea/vomiting	95 (43.8)	1.5 ± 0.7	33 (15.2)	1.2 ± 0.4	<0.001
Diarrhea	49 (22.6)	1.3 ± 0.6	24 (11.1)	1.2 ± 0.6	<0.001

Data are presented as number (%) and mean ± SD. AEFI = adverse event following immunization; SD = standard deviation. ^a^ *p* value analyzed by Wilcoxon signed rank test.

## Data Availability

The data presented in this study are available from the corresponding author upon reasonable request. The data are not publicly available due to restricted consent.

## Supplementary Materials

The following are available online at https://www.mdpi.com/article/10.3390/vaccines9101096/s1, Table S1: Solicited adverse events and grades, Table S2: Severity of adverse events following the first and second ChAdOx1 nCoV-19 vaccine doses by age group, Table S3: Incidence of the adverse events following the first and second ChAdOx1 nCoV-19 vaccine doses by healthcare workers under 30 years of age and over 30 years of age, Table S4: Patients who visited the outpatient clinic or emergency room due to adverse events following immunization.

## References

[1] World Health Organization WHO Coronavirus (COVID-19) Dashboard. https://covid19.who.int/.

[2] Korea Centers for Disease Control and Prevention COVID-19 Domestic Occurrence and Vaccination Status. https://ncv.kdca.go.kr/.

[3] Voysey M., Clemens S.A.C., Madhi S.A., Weckx L.Y., Folegatti P.M., Aley P.K., Angus B., Baillie V.L., Barnabas S.L., Bhorat Q.E. (2021). Safety and efficacy of the ChAdOx1 nCoV-19 vaccine (AZD1222) against SARS-CoV-2: An interim analysis of four randomised controlled trials in Brazil, South Africa, and the UK. Lancet.

[4] Ramasamy M.N., Minassian A.M., Ewer K.J., Flaxman A.L., Folegatti P.M., Owens D.R., Voysey M., Aley P.K., Angus B., Babbage G. (2020). Safety and immunogenicity of ChAdOx1 nCoV-19 vaccine administered in a prime-boost regimen in young and old adults (COV002): A single-blind, randomised, controlled, phase 2/3 trial. Lancet.

[5] Greinacher A., Thiele T., Warkentin T.E., Weisser K., Kyrle P.A., Eichinger S. (2021). Thrombotic Thrombocytopenia after ChAdOx1 nCov-19 Vaccination. N. Engl. J Med..

[6] AstraZeneca’s COVID-19 Vaccine: EMA Finds Possible Link to Very Rare Cases of Unusual Blood Clots with Low Blood Platelets. https://www.ema.europa.eu/en/news/astrazenecas-covid-19-vaccine-ema-finds-possible-link-very-rare-cases-unusual-blood-clots-low-blood.

[7] Aw J., Seng J.J.B., Seah S.S.Y., Low L.L. (2021). COVID-19 Vaccine Hesitancy—A Scoping Review of Literature in High-Income Countries. Vaccines.

[8] Oldenburg J., Klamroth R., Langer F., Albisetti M., von Auer C., Ay C., Korte W., Scharf R.E., Pötzsch B., Greinacher A. (2021). Diagnosis and Management of Vaccine-Related Thrombosis following AstraZeneca COVID-19 Vaccination: Guidance Statement from the GTH. Hamostaseologie.

[9] Korea Centers for Disease Control and Prevention Press Releases of COVID-19 Vaccination. http://ncov.mohw.go.kr/tcmBoardView.do?brdId=3&brdGubun=31&dataGubun=&ncvContSeq=5156&contSeq=5156&board_id=312&gubun=BDJ#.

[10] U.S. Food & Drug Administration Toxicity Grading Scale for Healthy Adult and Adolescent Volunteers Enrolled in Preventive Vaccine Clinical Trials. https://www.fda.gov/regulatory-information/search-fda-guidance-documents/toxicity-grading-scale-healthy-adult-and-adolescent-volunteers-enrolled-preventive-vaccine-clinical.

[11] Jeon M., Kim J., Oh C.E., Lee J.Y. (2021). Adverse Events Following Immunization Associated with Coronavirus Disease 2019 Vaccination Reported in the Mobile Vaccine Adverse Events Reporting System. J. Korean Med. Sci..

[12] Kim T., Park S.Y., Yu S., Park J.W., Lee E., Jeon M.H., Kim T.H., Choo E.J. (2021). Impacts of Side Effects to BNT162b2 and the First Dose of ChAdOx1 Anti-SARS-CoV-2 Vaccination on Work Productivity, the Need for Medical Attention, and Vaccine Acceptance: A Multicenter Survey on Healthcare Workers in Referral Teaching Hospitals in the Republic of Korea. Vaccines.

[13] Riad A., Pokorná A., Mekhemar M., Conrad J., Klugarová J., Koščík M., Klugar M., Attia S. (2021). Safety of ChAdOx1 nCoV-19 Vaccine: Independent Evidence from Two EU States. Vaccines.

[14] Hatmal M.M., Al-Hatamleh M.A.I., Olaimat A.N., Hatmal M., Alhaj-Qasem D.M., Olaimat T.M., Mohamud R. (2021). Side Effects and Perceptions Following COVID-19 Vaccination in Jordan: A Randomized, Cross-Sectional Study Implementing Machine Learning for Predicting Severity of Side Effects. Vaccines.

[15] Alhazmi A., Alamer E., Daws D., Hakami M., Darraj M., Abdelwahab S., Maghfuri A., Algaissi A. (2021). Evaluation of Side Effects Associated with COVID-19 Vaccines in Saudi Arabia. Vaccines.

[16] Kim S.H., Wi Y.M., Yun S.Y., Ryu J.S., Shin J.M., Lee E.H., Seo K.H., Lee S.H., Peck K.R. (2021). Adverse Events in Healthcare Workers after the First Dose of ChAdOx1 nCoV-19 or BNT162b2 mRNA COVID-19 Vaccination: A Single Center Experience. J. Korean Med. Sci..

[17] Folegatti P.M., Ewer K.J., Aley P.K., Angus B., Becker S., Belij-Rammerstorfer S., Bellamy D., Bibi S., Bittaye M., Clutterbuck E.A. (2020). Safety and immunogenicity of the ChAdOx1 nCoV-19 vaccine against SARS-CoV-2: A preliminary report of a phase 1/2, single-blind, randomised controlled trial. Lancet.

[18] Korea Centers for Disease Control and Prevention Weekly Report of Adverse Reaction after COVID-19 Vaccination (23th Week). https://ncv.kdca.go.kr/board.es?mid=a11707010000&bid=0032#content.

[19] Korea Centers for Disease Control and Prevention COVID-19 Vaccination Strategy and Implementation Plan of July. http://ncov.mohw.go.kr/tcmBoardView.do?brdId=3&brdGubun=31&dataGubun=&ncvContSeq=5626&contSeq=5626&board_id=312&gubun=ALL.

[20] Medicines & Healthcare Products Regulatory Agency Coronavirus Vaccine—Weekly Summary of Yellow Card Reporting. https://www.gov.uk/government/publications/coronavirus-covid-19-vaccine-adverse-reactions/coronavirus-vaccine-summary-of-yellow-card-reporting.

[21] Paul-Ehrlich-Institut Reports on Suspected Cases of Adverse Effects and Vaccination Complications Following a Vaccination for the Protection against COVID-19 (Reporting Period 27 December 2020 to 30 June 2021). https://www.pei.de/SharedDocs/Downloads/EN/newsroom-en/dossiers/safety-reports/safety-report-27-december-30-june-2021.pdf?__blob=publicationFile&v=6.

[22] Bunn C. (2021). ‘Getting a Clearer Picture’: Black Americans on the Factors That Overcame Their Vaccine Hesitancy. NBC News.

[23] Enwezor C.H., Peacock J.E., Seals A.L., Edelstein S.L., Hinkelman A.N., Wierzba T.F., Munawar I., Maguire P.D., Lagarde W.H., Runyon M.S. (2021). Changing Attitudes toward the COVID-19 Vaccine among North Carolina Participants in the COVID-19 Community Research Partnership. Vaccines.

[24] Cheng H., Peng Z., Luo W., Si S., Mo M., Zhou H., Xin X., Liu H., Yu Y. (2021). Efficacy and Safety of COVID-19 Vaccines in Phase III Trials: A Meta-Analysis. Vaccines.

