# Supplementary material for: Adverse Events Following Immunization Associated with the First and Second Doses of the ChAdOx1 nCoV-19 Vaccine among Healthcare Workers in Korea
Source: Vaccines (Basel). 2021 Sep 28;9(10):1096. doi: 10.3390/vaccines9101096 (PMC8537428; doi:10.3390/vaccines9101096)
Supplement: Supplementary file 1 [file vaccines-09-01096-s001.zip › vaccines-1312400-supplementary.pdf]

Adverse Events Following Immunization Associated with the first and second doses of the ChAdOx1 nCoV-19 vaccine among health care workers in Korea

ONLINE SUPPLEMENT MATERIALS

Table S1: Solicited adverse events and grades

Table S2: Severity of adverse events following the first and second dose of ChAdOx1 nCoV-19 vaccine by age group

Table S3: Incidence of the adverse events following the first and second doses of ChAdOx1 nCoV-19 vaccine by age under 30 years and over 30 years

Table S4: Patients who visited the outpatient clinic or emergency room due to adverse events following immunization

Table S1. Solicited adverse events and grades.

Adverse events	Grade 0	Grade 1	Grade 2	Grade 3	Grade 4
Injection site tenderness	None	Minor tenderness when injection site is touched	Very tender when injection site is touched, pain increased when it is moved	Severe pain so that limb movement reduced	-
Injection site resting pain	None	No interference with activity	Interferes with activity or requires use of pain-relieving medication	Prevents daily activity or requires use of prescription pain-relieving medication	-
Injection site redness[b]	None	< 2.5 cm	2.5–5 cm	5.1–10 cm	> 10 cm
Injection site swelling[b]	None	< 2.5 cm	2.5–5 cm	5.1–10 cm	> 10 cm
Fatigue	None	No interference with activity	Some interference with activity	Prevents daily activity	-
Headache	None	No interference with activity	Some interference with activity or requires use of pain relieving medication	Prevents daily activity or requires use of prescription pain-relieving medication	-
Malaise	None	No interference with activity	Some interference with activity or requires use of pain-relieving medication	Prevents daily activity or requires use of prescription pain-relieving medication	-
Arthralgia	None	No interference with activity	Some interference with activity or requires use of pain-relieving medication	Prevents daily activity or requires use of prescription pain-relieving medication	-
Chills	None	No interference with activity	Some interference with activity or requires medical intervention	Prevents daily activity or requires medical intervention	-
Fever[a]	< 38°C	38.0–38.4°C	38.5–38.9°C	39.0–40.0°C	> 40.0°C

Nausea/vomiting	None	No interference with activity or 1–2 episodes/day	Some interference with activity or ≥ 3 episodes/day	Prevents daily activity or requires outpatient intravenous hydration	-
Diarrhea	None	1 episode/day	2–3 episodes/day	4–5 episodes/day	≥ 6 episodes/day or requires outpatient intravenous hydration

[a]Measured value by using tympanic thermometer; [b]Measured the largest single diameter of lesion.

Table S2. Severity of adverse events following the first and second dose of ChAdOx1 nCoV-19 vaccine by age group.

**Adverse events**	**1st dose**				**2nd dose**			
	**< 30 yr (*n* = 394)**	**30–50 yr (*n* = 471)**	**> 50 yr (*n* = 129)**	***p* value**	**< 30 yr (n =244)**	**30–50yr (n =378)**	**> 50 yr (n =105)**	***p* value**
ALL				0.011				0.466
None	5 (1.3)	7 (1.5)	7 (5.4)		21 (8.6)	32 (8.5)	13 (12.4)	
Grade 1	19 (4.8)	56 (11.9)	33 (25.6)		70 (28.7)	105 (27.8)	46 (43.8)	
Grade 2	165 (41.9)	201 (42.7)	56 (43.4)		114 (46.7)	159 (42.1)	30 (28.6)	
Grade 3	199 (50.5)	196 (41.6)	33 (25.6)		38 (15.6)	80 (21.2)	16 (15.2)	
Grade 4	6 (1.5)	11 (2.3)	0 (0.0)		1 (0.4)	2 (0.5)	0 (0.0)	
Local AEFIs				<0.001				0.079
None	12 (3.0)	21 (4.5)	16 (12.4)		39 (16.0)	52 (13.8)	24 (22.9)	
Grade 1	44 (11.2)	99 (21.0)	48 (37.2)		81 (33.2)	117 (31.0)	44 (41.9)	
Grade 2	182 (46.2)	187 (39.7)	36 (27.9)		91 (37.3)	139 (36.8)	22 (21.0)	
Grade 3	151 (38.3)	157 (33.3)	29 (22.5)		32 (13.1)	68 (18.0)	15 (14.3)	
Grade 4	5 (1.3)	7 (1.5)	0 (0.0)		1 (0.4)	2 (0.5)	0 (0.0)	
Systemic AEFIs				<0.001				0.711
None	11 (2.8)	19 (4.0)	16 (12.4)		62 (25.4)	88 (23.3)	28 (26.7)	
Grade 1	50 (12.7)	97 (20.6)	42 (32.6)		88 (36.1)	153 (40.5)	53 (50.5)	
Grade 2	229 (58.1)	254 (53.9)	64 (49.6)		87 (35.7)	113 (29.9)	23 (21.9)	
Grade 3	103 (26.1)	97 (20.6)	7 (5.4)		7 (2.9)	24 (6.3)	1 (1.0)	
Grade 4	1 (0.3)	4 (0.8)	0 (0.0)		0 (0.0)	0 (0.0)	0 (0.0)	
Tenderness				<0.001				0.051
None	12 (3.0)	23 (4.9)	20 (15.5)		46 (18.9)	54 (14.3)	25 (23.8)	
Grade 1	48 (12.2)	109 (23.1)	51 (39.5)		75 (30.7)	120 (31.7)	45 (42.9)	
Grade 2	187 (47.5)	182 (38.6)	31 (24.0)		91 (37.3)	138 (36.5)	21 (20.0)	
Grade 3	147 (37.3)	157 (33.3)	27 (20.9)		32 (13.1)	66 (17.5)	14 (13.3)	
Resting pain				<0.001				0.337
None	39 (9.9)	47 (10.0)	33 (25.6)		87 (35.7)	119 (31.5)	40 (38.1)	
Grade 1	206 (52.3)	267 (56.7)	70 (54.3)		138 (56.6)	217 (57.4)	58 (55.2)	
Grade 2	147 (37.3)	156 (33.1)	25 (19.4)		19 (7.8)	42 (11.1)	7 (6.7)	
Grade 3	2 (0.5)	1 (0.2)	1 (0.8)		0 (0.0)	0 (0.0)	0 (0.0)	
Redness[a]				0.129				0.071
None	249 (63.2)	312 (66.2)	94 (72.9)		193 (79.1)	268 (70.9)	76 (72.4)	
Grade 1	111 (28.2)	113 (24.0)	27 (20.9)		47 (19.3)	98 (25.9)	27 (25.7)	
Grade 2	15 (3.8)	31 (6.6)	3 (2.3)		4 (1.6)	7 (1.9)	1 (1.0)	
Grade 3	16 (4.1)	10 (2.1)	5 (3.9)		0 (0.0)	4 (1.1)	1 (1.0)	
Grade 4	3 (0.8)	5 (1.1)	0 (0.0)		0 (0.0)	1 (0.3)	0 (0.0)	
Swelling[a]				0.051				0.364
None	185 (47.0)	250 (53.1)	75 (58.1)		172 (70.5)	249 (65.9)	67 (63.8)	
Grade 1	136 (34.5)	146 (31.0)	44 (34.1)		57 (23.4)	99 (26.2)	30 (28.6)	
Grade 2	45 (11.4)	56 (11.9)	7 (5.4)		13 (5.3)	24 (6.3)	5 (4.8)	
Grade 3	25 (6.3)	12 (2.5)	3 (2.3)		1 (0.4)	4 (1.1)	3 (2.9)	
Grade 4	3 (0.8)	7 (1.5)	0 (0.0)		1 (0.4)	2 (0.5)	0 (0.0)	
Fatigue				<0.001				0.168
None	20 (5.1)	31 (6.6)	20 (15.5)		81 (33.2)	104 (27.5)	37 (35.2)	
Grade 1	92 (23.4)	133 (28.2)	51 (39.5)		95 (38.9)	164 (43.4)	51 (48.6)	
Grade 2	187 (47.5)	215 (45.6)	52 (40.3)		61 (25.0)	88 (23.3)	16 (15.2)	
Grade 3	95 (24.1)	92 (19.5)	6 (4.7)		7 (2.9)	22 (5.8)	1 (1.0)	
Headache				<0.001				0.694
None	60 (15.2)	115 (24.4)	47 (36.4)		140 (57.4)	229 (60.6)	64 (61.0)	

Grade 1	89 (22.6)	127 (27.0)	46 (35.7)		54 (22.1)	92 (24.3)	29 (27.6)	
Grade 2	243 (61.7)	223 (47.3)	36 (27.9)		50 (20.5)	56 (14.8)	12 (11.4)	
Grade 3	2 (0.5)	6 (1.3)	0 (0.0)		0 (0.0)	1 (0.3)	0 (0.0)	
Malaise				<0.001				0.281
None	51 (12.9)	74 (15.7)	36 (27.9)		156 (63.9)	218 (57.7)	61 (58.1)	
Grade 1	89 (22.6)	109 (23.1)	38 (29.5)		56 (23.0)	105 (27.8)	35 (33.3)	
Grade 2	247 (62.7)	279 (59.2)	54 (41.9)		32 (13.1)	53 (14.0)	9 (8.6)	
Grade 3	7 (1.8)	9 (1.9)	1 (0.8)		0 (0.0)	2 (0.5)	0 (0.0)	
Arthralgia				0.086				0.045
None	154 (39.1)	169 (35.9)	60 (46.5)		197 (80.7)	293 (77.5)	72 (68.6)	
Grade 1	82 (20.8)	114 (24.2)	31 (24.0)		30 (12.3)	61 (16.1)	23 (21.9)	
Grade 2	155 (39.3)	184 (39.1)	38 (29.5)		17 (7.0)	24 (6.3)	10 (9.5)	
Grade 3	3 (0.8)	4 (0.8)	0 (0.0)		0 (0.0)	0 (0.0)	0 (0.0)	
Chills				0.001				0.570
None	117 (29.7)	149 (31.6)	60 (46.5)		206 (84.4)	308 (81.5)	89 (84.8)	
Grade 1	90 (22.8)	109 (23.1)	33 (25.6)		23 (9.4)	50 (13.2)	13 (12.4)	
Grade 2	179 (45.4)	207 (43.9)	35 (27.1)		15 (6.1)	17 (4.5)	3 (2.9)	
Grade 3	8 (2.0)	6 (1.3)	1 (0.8)		0 (0.0)	3 (0.8)	0 (0.0)	
Fever[b]				<0.001				0.084
None	264 (67.0)	337 (71.5)	119 (92.2)		243 (99.6)	367 (97.1)	103 (98.1)	
Grade 1	83 (21.1)	88 (18.7)	10 (7.8)		1 (0.4)	8 (2.1)	2 (1.9)	
Grade 2	40 (10.2)	34 (7.2)	0 (0.0)		0 (0.0)	2 (0.5)	0 (0.0)	
Grade 3	7 (1.8)	10 (2.1)	0 (0.0)		0 (0.0)	1 (0.3)	0 (0.0)	
Grade 4	0 (0.0)	2 (0.4)	0 (0.0)		0 (0.0)	0 (0.0)	0 (0.0)	
Nausea/vomiting				<0.001				0.446
None	217 (55.1)	311 (66.0)	104 (80.6)		208 (85.2)	333 (88.1)	94 (89.5)	
Grade 1	143 (36.3)	124 (26.3)	21 (16.3)		32 (13.1)	41 (10.8)	11 (10.5)	
Grade 2	28 (7.1)	35 (7.4)	4 (3.1)		3 (1.2)	3 (0.8)	0 (0.0)	
Grade 3	6 (1.5)	1 (0.2)	0 (0.0)		1 (0.4)	1 (0.3)	0 (0.0)	
Diarrhea				0.081				0.184
None	302 (76.6)	386 (82.0)	108 (83.7)		219 (89.8)	333 (88.1)	99 (94.3)	
Grade 1	56 (14.2)	54 (11.5)	9 (7.0)		9 (3.7)	28 (7.4)	6 (5.7)	
Grade 2	30 (7.6)	26 (5.5)	10 (7.8)		14 (5.7)	15 (4.0)	0 (0.0)	
Grade 3	5 (1.3)	3 (0.6)	2 (1.6)		2 (0.8)	2 (0.5)	0 (0.0)	
Grade 4	1 (0.3)	2 (0.4)	0 (0.0)		0 (0.0)	0 (0.0)	0 (0.0)	

Values are presented as number (%). AEFI = adverse event following immunization. [a]Measured the largest single diameter of lesion; [b]Measured value by using tympanic thermometer.

**AEFIs**		A vs. B	A vs. C	B vs. C
**1st dose**	Any AEFIs		0.012	0.016
	Local AEFIs		< 0.001	0.002
	Systemic AEFIs		< 0.001	0.001
	Tenderness		< 0.001	< 0.001
	Resting pain		< 0.001	< 0.001
	Fatigue		< 0.001	0.002
	Headache	< 0.001	< 0.001	0.007
	Malaise		< 0.001	0.002
	Chills		0.001	0.002
	Fever		< 0.001	< 0.001
	Nausea/vomiting	0.001	< 0.001	0.002
2nd dose	Arthralgia		0.011	

Post-hoc analysis by Bonferroni correction. A, age < 30 yr; B, age 30–50 yr; C, age > 50 yr. AEFI = adverse event following immunization.

Table S3. Incidence of the adverse events following the first and second doses of ChAdOx1 nCoV-19 vaccine by age under 30 years and over 30 years.

	1st dose			2nd dose		
Adverse events	< 30 yr (*n* = 394)	≥ 30 yr (*n* = 600)	*p* value	< 30 yr (*n* = 244)	≥ 30 yr (*n* = 483)	*p* value
Any AEFIs	389 (98.7)	586 (97.7)	0.336	223 (91.4)	438 (90.7)	0.859
Local AEFIs	382 (97.0)	563 (93.8)	0.038	205 (84.0)	407 (84.3)	> 0.999
Tenderness	382 (97.0)	557 (92.8)	0.008	198 (81.1)	404 (83.6)	0.460
Resting pain	355 (90.1)	520 (86.7)	0.126	157 (64.3)	324 (67.1)	0.514
Redness	145 (36.8)	194 (32.3)	0.166	51 (20.9)	139 (28.8)	0.028
Swelling	209 (53.0)	275 (45.8)	0.031	72 (29.5)	167 (34.6)	0.197
Systemic AEFIs	383 (97.2)	565 (94.2)	0.038	182 (74.6)	367 (76.0)	0.748
Fatigue	374 (94.9)	549 (91.5)	0.054	163 (66.8)	342 (70.8)	0.307
Headache	334 (84.8)	438 (73.0)	< 0.001	104 (42.6)	190 (39.3)	0.440
Malaise	343 (87.1)	490 (81.7)	0.030	88 (36.1)	204 (42.2)	0.128
Arthralgia	240 (60.9)	371 (61.8)	0.822	47 (19.3)	118 (24.4)	0.140
Chills	277 (70.3)	391 (65.2)	0.106	38 (15.6)	86 (17.8)	0.515
Fever	130 (33.0)	144 (24.0)	0.002	1 (0.4)	13 (2.7)	0.068
Nausea/vomiting	177 (44.9)	185 (30.8)	< 0.001	36 (14.8)	56 (11.6)	0.275
Diarrhea	92 (23.4)	106 (17.7)	0.035	25 (10.2)	51 (10.6)	0.998

Data are presented as number (%). AEFI = adverse event following immunization.

Table S4. Patients who visited the outpatient clinic or emergency room due to adverse events following immunization.

No.	Age	Sex	Chief complaint	Dose	Time to visit after vaccination	Medical care unit
1	24	Female	pain, redness, warmth at the injection site	1st	Day 6	ID
2	51	Female	Redness, warmth, itching sense at the injection site	1st	Day 5	ID
3	26	Female	Urticaria at both arms	1st	Day 1	ID
4	27	Female	Urticaria at both legs	1st	Day 2	ER
5	31	Female	Fever	1st	Day 2	ER
6	45	Female	Fever, chills, malaise	1st	Day 2	ER
7	19	Female	Chills, headache, malaise, nausea	1st	Day 1	ID
8	22	Female	Hypotension, nausea, dyspnea, dizziness	1st	Day 0	ER
9	39	Female	Chills, malaise	1st	Day 1	ER
10	23	Female	Dizziness, nausea, weakness, and tingling sense of lower extremities	1st	Day 0	ER
11	24	Female	Pain, redness, swelling, induration, itching sense at the injection site	1st	Day 7	ID
12	30	Male	Diarrhea	1st	Day 2	ID
13	37	Male	Diarrhea, nausea, vomiting, malaise, fatigue	1st	Day 2	ID
14	27	Female	Throat pain and swelling	2nd	Day 1	ID
15	46	Female	Headache, chills, fatigue	2nd	Day 3	ID
16	23	Female	Petechiae on both legs	2nd	Day 3	ID
17	65	Male	Multiple ecchymosis on both arms	2nd	Day 7	ID
18	38	Female	Arthralgia, fatigue	2nd	Day 7	ID

ID = department of infectious disease, ER = emergency room.
